# The involvement of serum exosomal miR-500-3p and miR-770-3p in aging: modulation by calorie restriction

**DOI:** 10.18632/oncotarget.23651

**Published:** 2017-12-24

**Authors:** Eun Kyeong Lee, Hyoung Oh Jeong, Eun Jin Bang, Chul Hong Kim, Ji Young Mun, Seulgi Noh, Jeong-An Gim, Dae Hyun Kim, Ki Wung Chung, Byung Pal Yu, Hae Young Chung

**Affiliations:** ^1^ Molecular Inflammation Research Center for Aging Intervention, College of Pharmacy, Pusan National University, Busan, Republic of Korea; ^2^ Genomictree Inc., Daejeon, Republic of Korea; ^3^ Department of Biomedical Laboratory Science, College of Health Science, Eulji University, Seongnam-Si, Gyeonggi-Do, Republic of Korea; ^4^ BK21 Plus Program, Department of Senior Healthcare, Graduate School, Eulji University, Seongnam-Si, Gyeonggi-Do, Republic of Korea; ^5^ Department of Biological Sciences, College of Natural Sciences, Pusan National University, Busan, Republic of Korea; ^6^ Department of Physiology, The University of Texas Health Science Center at San Antonio, San Antonio, TX, USA

**Keywords:** aging, calorie restriction, exosome, miR-500-3p, miR-770-3p, Gerotarget

## Abstract

Recent studies have shown a role for miRNAs in aging and age-related diseases, and the modulation of miRNA expression by diet attracts attention as a new therapeutic strategy. Here, we focused on identifying specific exosomal miRNAs derived from serum of aged rats and the effect of short-term calorie restriction (CR) on their expression.

Exosomes from serum of young (7-month), old (22-month), and old-CR Sprague Dawley rats were isolated and characterized by transmission electron microscopy analyses, dynamic light scattering measurements, and Western blotting. A total of 12 significantly expressed miRNAs in serum exosomes of young and old rats were identified by next generation sequencing. After analysis of qRT-PCR, we found that miR-500-3p and miR-770-3p expression was significantly upregulated by aging and downregulated by CR. Furthermore, receiver operating characteristic (ROC) curve revealed that the selected miRNAs represented high accuracy in discriminating old rats from young rats. Finally, PANTHER analysis predicted selected miRNAs targets genes involved in Wnt/chemokines and cytokines -related inflammatory signaling pathway and function as transcription factor.

In conclusion, our results suggest that the expression of serum exosomal miR-500-3p and miR-770-3p was significantly increased with aging, whereas these were decreased by CR, and age-/CR-modulated exosomal miR-500-3p and miR-770-3p could potentially be used as informative biomarkers candidates for aging.

## INTRODUCTION

Exosomes are extracellular vesicles (40-120 nm) released from various cell types, e.g. stem cells, primary cells of the immune and nervous systems, as well as several tumor cells [1]. They were first discovered from studies of recycling transferrin receptor during maturation of reticulocytes into erythrocytes [2]. For many years, exosomes were considered as means for cells to shed excess proteins. Recent studies have shown attractive results for exosomes as new candidates with important roles in intercellular and tissue-level communication through protein, mRNA, and miRNA delivery. The discovery of exosomal miRNAs has especially allowed studies on their possible role as biomarkers or therapeutic targets in various diseases [3].

MicroRNAs (miRNAs) are non-coding RNAs of approximately 18-25 nucleotides in length, which regulate protein expression at the post-transcriptional level by binding to the 3′-UTR region of their target mRNAs [4]. It is estimated that approximately 60% of human genes are regulated by miRNAs, suggesting that miRNAs are important regulators of most physiological and pathological processes [5]. miRNA is growing as potential pathological biomarker candidates as it is more stable than mRNAs [6] and is easily accessible in circulating body fluid such as blood, saliva, and urine [7]. Additionally, it has been associated with regulating both activation and resolution of inflammation through modulation of aging-related networks such as insulin, nuclear factor-κB (NF-κB), and p53 signaling pathways [8].

CR is the most well-known and scientifically accepted way to increase life span [9]. The detailed mechanism of CR is not known, but both short-term and long-term CR reduce extent of age-related decline and damage in overall tissue function, and delay and reserve the gene expression patterns associated with regulation of aging and longevity [10]. In our previous reports, very short-term CR (10 days) attenuated renal inflammation through inhibition of reactive oxygen species-induced NF-κB and activator protein 1 (AP-1) [11]. Moreover, short-term CR (4 weeks) ameliorated age-related aberrant methylation patterns at promoter regions [12]. Besides, Dhahbi (2014) reported that CR regulates circulating miRNA expression pattern during the aging process [13].

Here, to investigate the exosomal miRNAs in serum from young and old rats, we performed small RNA deep sequencing and examined the effects of short-term CR on miRNA signatures in old rats. We found that among analyzed miRNAs, miR-500-3p and miR-770-3p were modulated by aging and CR. Furthermore, we analyzed the role of miR-500-3p and miR-770-3p in aging using PANTHER, a bioinformatics software. The findings from our study provide the notion that miR-500-3p and miR-770-3p in serum exosomes of aging rats might be potential candidates to understand aging and CR.

## RESULTS

### Purification and characterization of exosomes derived from serum of young, old, and old-CR rats

**Figure 1:**
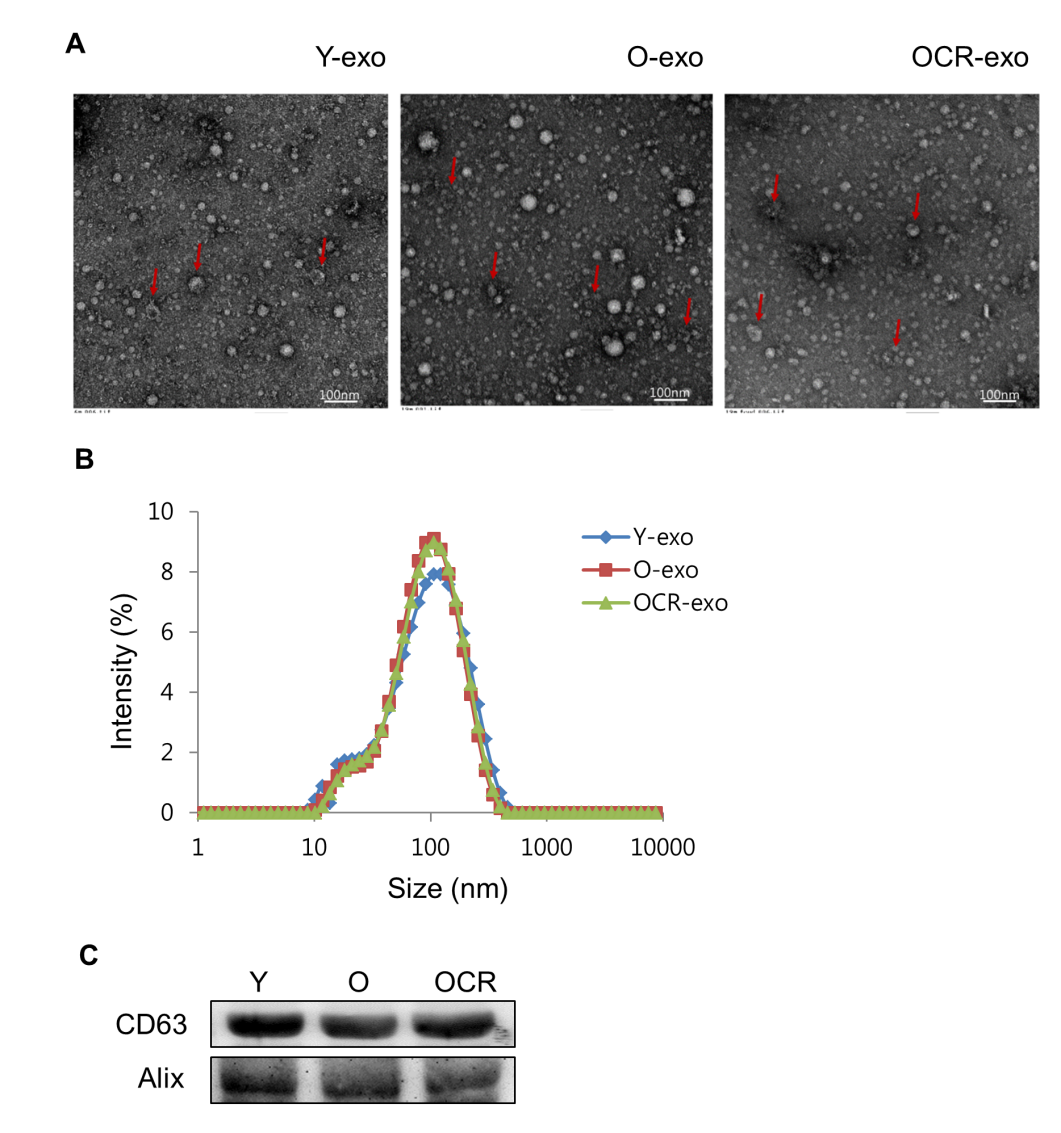
Characterization of exosomes purified from serum of young, old, and old-CR rats (**A**) Exosomes were visualized by transmission electron microscopy. The arrow indicates the exosome with cup-shaped morphology. (**B**) Size analysis of exosomes extracted from the young (Y-exo), old (O-exo), and old-CR (OCR-exo) rats through DLS measurements. (**C**) Western blotting for CD63 and Alix as exosomal markers. Y, young; O, old; OCR, old-CR.

To confirm that purified vesicles were exosomes, we first examined the morphology of the vesicles by transmission electron microscopy (TEM). As shown in Figure 1A, the vesicles revealed round- or cup-shaped structures with various sizes. Additionally, dynamic light scattering (DLS) was used to measure the size of exosomal vesicles. Its average size was 100 nm and there was no difference in the size of exosomes according to the aging and diet in all the groups of rats (Figure 1B). Finally, Western blotting analysis was performed to evaluate the expression of exosomal surface markers in purified vesicles. The exosomal markers, CD63 and Alix, were present in vesicles derived from each group (Figure 1C). Taken together, purified exosomes were fully consistent with the morphology and sizes reported previously [14].

### miRNA profiles of exosomes derived from young and old rats

**Figure 2:**
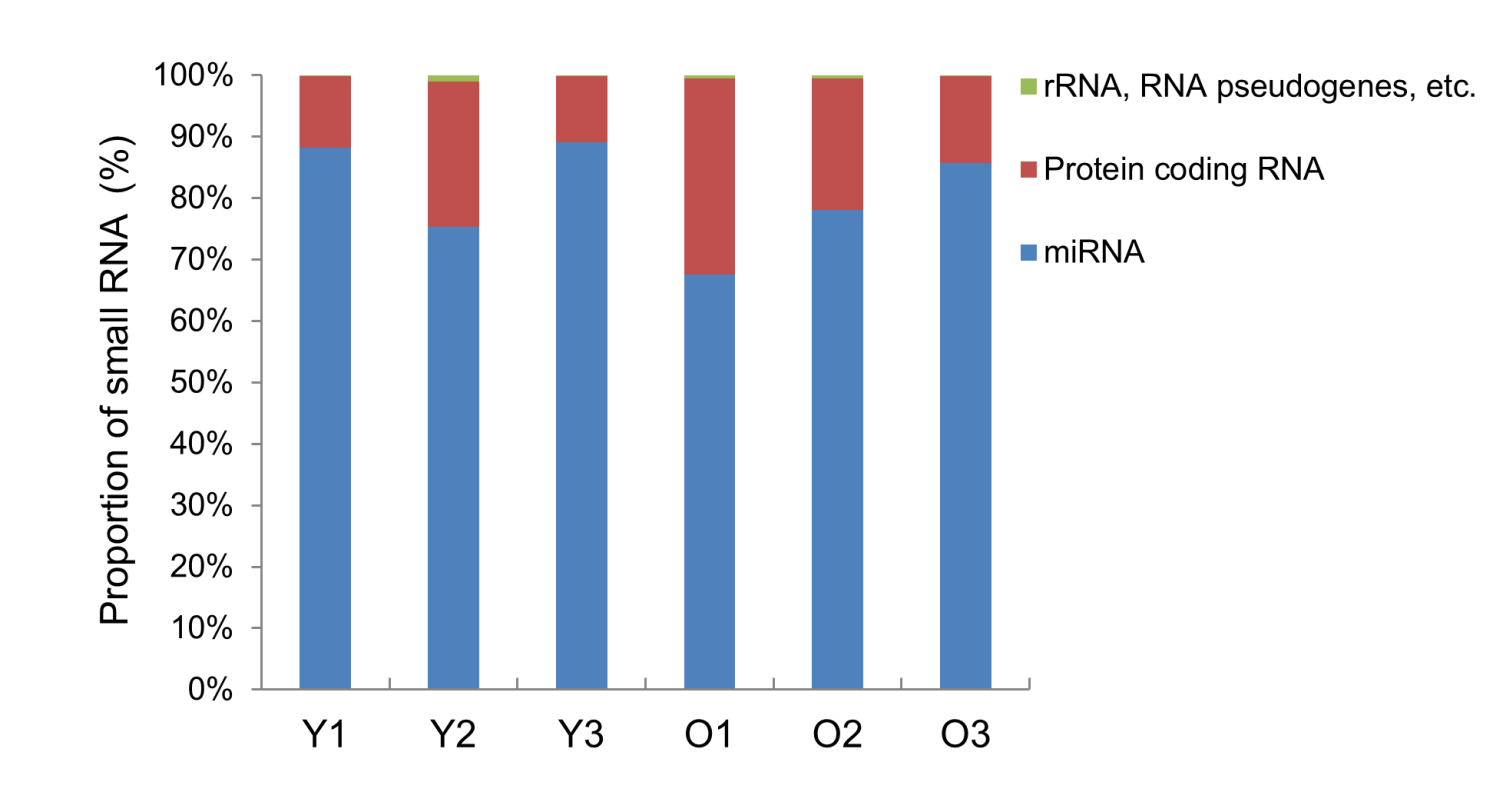
Bar graph of small RNA classification in the exosomes The distribution of RNA classes in exosomes purified from young (Y) and old (O) rats is shown as a percentage of the total annotated small RNAs.

To investigate the effects of aging on exosomal miRNA expression, we performed small RNA sequencing to analyze the miRNA levels in exosomes purified from the young and old rats. An average of 10.4 ± 3.4 and 8.0 ± 3.0 ng small RNAs were obtained from exosomes purified from the young and old rats, respectively. After performing RNA sequencing, we checked that the small RNA constituted over 80% of the total reads in the exosomes from young and old rats (data not shown) and then used the obtained data for mapping and we observed a proportion of small RNAs in the exosomes. A large proportion of the small RNAs in exosomes were annotated as miRNAs (70%), and approximately 10% of sequences were protein-coding RNAs. Also, rRNA, RNA pseudogenes, processed pseudogenes, processed transcripts, and lncRNAs formed below 1% of small RNA in exosomes from young and old rats (Figure 2). Next, we selected miRNAs with cutoff-values of greater than 3-fold change with *P* < 0.05 for the old rat group using the young rat group as a reference, and obtained 12 miRNAs which were significantly changed in their expression levels based on RNA-sequencing analysis. Of 12 miRNAs, 8 were upregulated and 4 were downregulated in the old group compared to those in the young group (Table 1).

**Table 1:** Genes regulated by more than 3-fold change in NGS analysis

Old vs. Young					
Up-regulated			Down-regulated		
Name	FC	p-value	Name	FC	p-value
rno-miR-500-3p	223	0.0059	rno-miR-450a-5p	292	0.0159
rno-miR-770-3p	165	0.0491	rno-miR-196c-3p	140	0.0185
rno-miR-6324	161	0.0028	rno-miR-34b-3p	4	0.0041
rno-miR-455-3p	132	0.0124	rno-miR-10a-3p	3	0.0374
rno-miR-487b-3p	84	0.0309			
rno-miR-26b-3p	83	0.0297			
rno-miR-127-3p	17	0.0232			
rno-miR-148b-3p	4	0.0358			

FC, fold change

### Validation of exosomal miRNA expression

**Figure 3:**
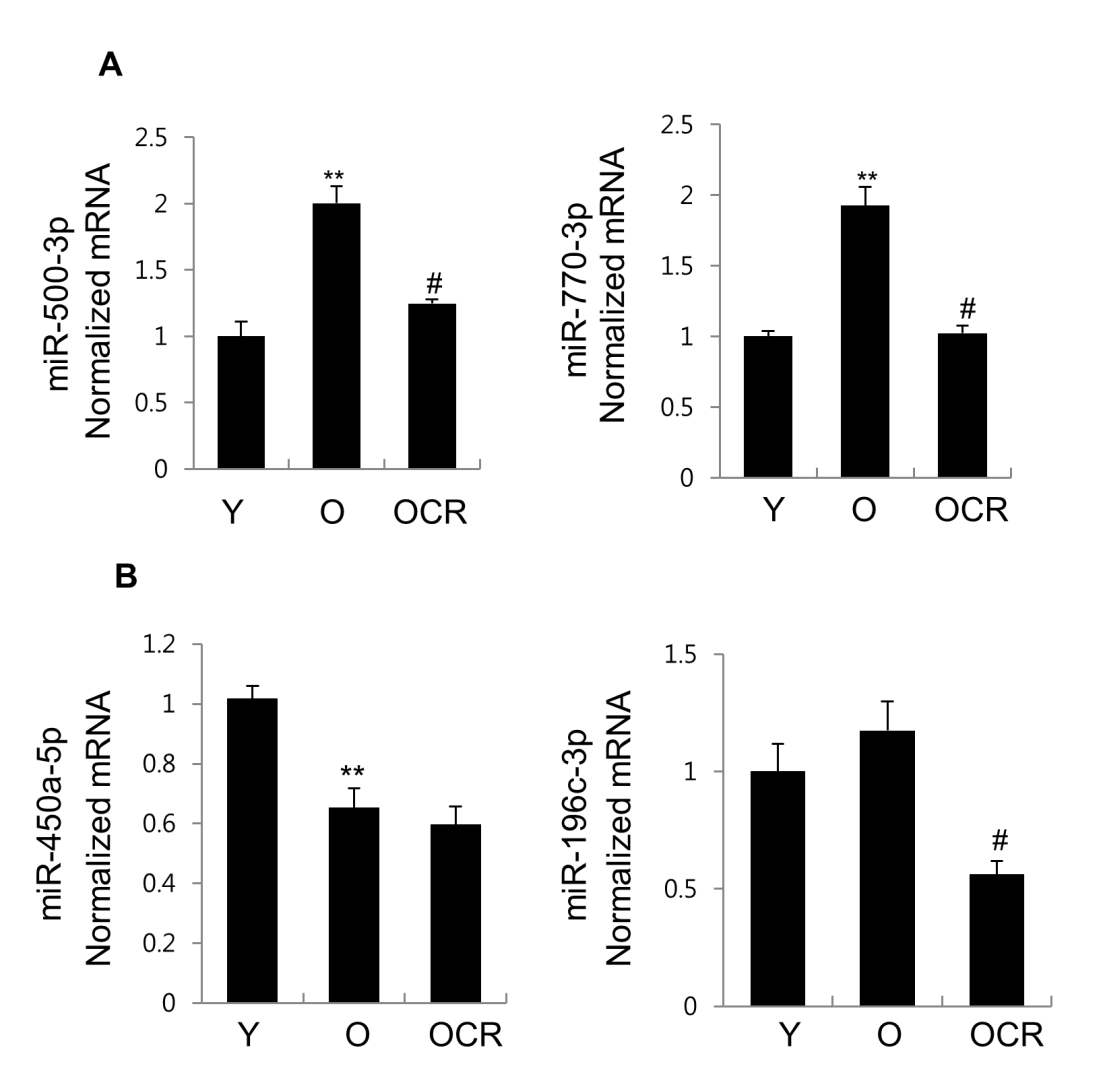
Expression of 4 miRNAs in exosomes purified from young, old, and old-CR rats qRT-PCR was used to investigate the expression of (**A**) miR-500-3p, miR-770-3p, (**B**) miR-450a-5p, and miR-196c-3p. **B.** The obtained values were normalized to cel-miR-54 as an internal control. Each value is the mean ± S.E. ** *P* < 0.01 *vs*. young rats group; ^#^*P* < 0.05 *vs*. old rats group (*n* = 6). Y, young; O, old; OCR, old-CR.

To verify the reliability of the miRNA sequencing results, the most upregulated (miR-500-3p and miR-770-3p) and downregulated (miR-450a-5p and miR-196c-3p) miRNAs in the exosomes derived from old rats in comparison to those from young rats, were selected for further verification using quantitative real-time PCR (qRT-PCR). Figure 3A showed that the expression of miR-500-3p and miR-770-3p was significantly increased in the old group compared to that in the young group. On the other hand, with the downregulated miRNAs in the exosomes, in old group compared to young group, miR-450a-5p expression decreased, but miR-196c-3p expression did not show any significant change (Figure 3B).

Furthermore, CR effect on these miRNAs expression was also examined using qRT-PCR method. The result showed significant downregulation of miR-500-3p and miR-770-3p, which was significantly increased in old rats in comparison to young rats. In contrast, miR-450a-5p, which was significantly downregulated in old rats, did not show CR effect. Whereas miR-196c-3p expression was decreased by CR (Figure 3A and 3B). Therefore, Figure 3 demonstrates that the qRT-PCR results for miR-500-3p, miR-770-3p, and miR-450a-5p were consistent with those of the RNA sequencing data. miR-500-3p and miR-770-3p, that were significantly changed in both aging and diet, were selected for further analysis.

In addition, we have examined the expression of miR-500-3p and miR-770-3p in serum of young, old, and old-CR rats. The results showed that expression of two miRNAs in serum was not significantly changed in accordance to both aging and diet (Supplementary Figure 1). Therefore, it suggests that miR-500-3p and miR-770-3p possess exosome specificity.

### Exosomal miR-500-3p and miR-770-3p as candidates for aging

**Figure 4:**
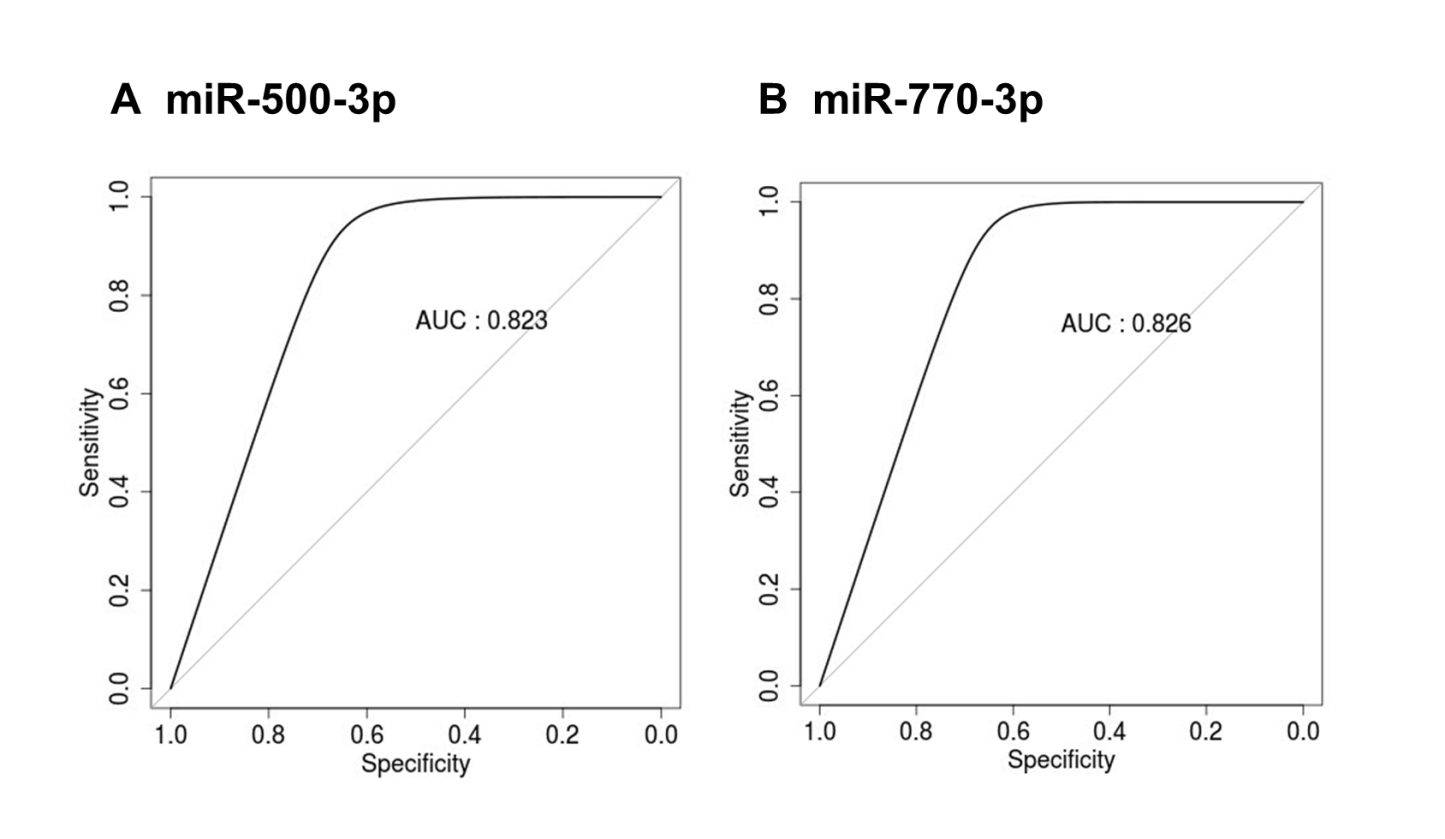
ROC curve analysis of miR-500-3p and miR-770-3p in the serum exosomes of young and old rats ROC curves for classifying the serum exosomal miRNAs from young and old rats were produced using the expression values for (**A**) miR-500-3p and (**B**) miR-770-3p. ROC, receiver operating characteristic; AUC, area under the curve.

To evaluate potential possibility for these miRNAs to be utilized as candidates for aging, the receiver operating characteristic (ROC) curves were constructed for miR-500-3p and miR-770-3p (Figure 4). The area under the curve (AUC) for miR-500-3p and miR-770-3p were 0.823 and 0.826, respectively. These data suggests that exosomal miR-500-3p and miR-770-3p could be used to discriminate old rats from young rats.

### Target genes and functional prediction of miR-500-3p and miR-770-3p

To understand more thoroughly how miR-500-3p and miR-770-3p may contribute to the aging and CR, we predicted their target genes using TargetScan and analyzed the biological processes, pathways, and protein classes associated with each of the miRNAs using the PANTHER database. From the PANTHER analysis, we found that most of their predicted functions such as the biological processes, pathways, and protein classes appears to be similar between two miRNAs (Table 2). Particularly, both miR-500-3p and miR-770-3p could mainly target the transcription factors that control the gene transcription and genes involved in the Wnt/chemokines and cytokines-related inflammatory signaling pathways. This analysis suggests that miR-500-3p and miR-770-3p may be involved in similar biological functions in aging process.

**Table 2:** Enrichment analysis for biological processes, pathways, and protein classes of target genes regulated by miR-500-3p and miR-770-3p

	Biological process	#	Pathway	#	Protein Class	#
miR-500-3p	Cellular process	73	Wnt signaling	7	Nucleic acid binding	24
	Metabolic process	59	Integrin signaling	4	Transcription factor	23
	Developmental process	26	Gonadotropin-releasing hormone receptor	4	Enzyme modulator	15
	Biological regulation	21	Angiogenesis	3	Hydrolase	11
	Response to stimulus	16	Inflammation mediated by chemokine and cytokine	3	Transferase	10
miR-770-3p	Cellular process	898	Wnt signaling	45	Nucleic acid binding	254
	Metabolic process	668	Gonadotropin-releasing hormone receptor	44	Transcription factor	177
	Developmental process	275	Integrin signaling	33	Transferase	164
	Localization	252	Angiogenesis	31	Enzyme modulator	162
	Biological regulation	251	Inflammation mediated by chemokine and cytokine	31	Receptor	143

The top five pathways for each parameter are listed and where applicable, the number of genes (#) for the particular pathway is included.

## DISCUSSION

This study aims to identify exosomal miRNAs which were modulated by aging and CR and to predict their biological roles in aging. Using next-generation sequencing, we determined that miRNAs constituted almost the entire small RNAs existing in exosomes, and analysis of miRNA expression profiles showed 8 upregulated miRNAs and 4 downregulated miRNAs with aging. Among identified miRNAs, miR-500-3p and miR-770-3p, the most highly upregulated miRNAs with aging were significantly decreased by CR. Furthermore, ROC analysis was confirmed by high sensitivities of selected exosomal miRNAs as candidates for aging. Finally, using PANTHER analysis, we identified that both miR-500-3p and miR-770-3p might control the gene transcription through regulation of transcription factors and Wnt/chemokines and cytokines-related inflammation signaling pathways during aging. Therefore, expression of exosomal miR-500-3p and miR-770-3p in aged rat serum might present further possibilities as potential candidates for investigating the aging process.

Exosomes contain various molecular constituents according to cell origin, including protein, lipids and nucleotides. This exosomal characteristic makes it possible to use exosomes as biomarkers for pathophysiological conditions [3]. Particularly, specific exosomal miRNA levels in body fluid have been associated with aging and age-related inflammatory disease [8]. Circulating exosomal miR-21 has been reported as a biomarker in each tumor stage of colorectal cancer [15] and plasma exosomal miR-23b-3p, miR-10b-5p and miR-21-5p have been reported as prognostic biomarkers for non-small-cell lung cancer [16]. Balkom et al., (2013) showed that endothelial cells secrete exosomes containing miR-214, which suppress senescence and stimulates an angiogenetic program in target cell [17]. However, exosomal miR-214 expression in our study was not affected by age and CR (data not shown). In addition, Machida et al., (2015) examined exosomal miRNAs profiles in salivary using microarray analysis and have reported that miR-24-3p is significantly increased in human salivary from older individuals compared to those in younger individuals, and miR-24-3p could correlate with periodontal inflammation by age-related decline in salivary function [18]. These previous reports do not show similar results with serum exosomal miRNAs profiles obtained in our study, suggesting that these miRNAs may possess cells or tissue specificity.

Previous studies have shown that the sequence of many miRNAs is found to be highly evolutionarily conserved, in their mature form, among different organisms, although there are significant genomic differences between human and rat [19]. Aranha et al. [20] showed that miR-16, let-7a and miR-34a, whose expression patterns are conserved in mouse, rat and human neural differentiation, are involved in mammalian neuronal development. Also, Gutiérrez-Escolano et al. [21] showed that plasma miRNAs, miR-30a, miR-30c, and miR-30e, were significantly higher in both contrast-induced nephropathy (CIN) rats and CIN patients when compared with those in non-CIN control, and might serve as biomarkers for CIN. Additionally, Zhang et al. [22] have demonstrated miR-29b and miR-142-5p that was changed significantly in human serum during aging process, and we have confirmed that these two miRNAs also showed same expression pattern in young and aged rat serum (Supplementary Figure 2). Therefore, we may need further investigation on miR-500-3p and miR-770-3p expression in human serum exosome to strengthen their potential role as aging biomarkers.

There are limited previous published reports on miR-500-3p and miR-770-3p which were found in our study. miR-500-3p expression was significantly downregulated in cardiac tissues of 7-day-old mice compared to 1-and 6-day-old mice, which may have important implications for the myocardial regenerative process [23]. Also, miR-500-3p showed a significantly increased expression in peripheral CD3^+^ T lymphocytes from susceptible mouse strain to collagen-induced arthritis (CIA) and these results imply miRNA that could be involved in inflammatory process following CIA induction [24]. There are no previously published reports on miR-770-3p. Therefore, in order to better understand the potential role of these two miRNAs during aging, we performed PANTHER analysis, and found that both these miRNAs might control the gene transcription through targeting of transcription factors such as *YY1, FOXO1, PPARD* and Wnt/chemokines and cytokines related-inflammation signaling pathways.

Wnt signaling plays an important role in cell proliferation, differentiation, cell fate determination, apoptosis, and stem cell renewal [25]. However, Wnt signaling activation is associated with aging process and age-related diseases. Under conditions of oxidative stress, canonical Wnt activation results in cell cycle exit and senescence through interaction between β-catenin and forkhead box O (FOXO) in the nucleus [26]. In addition, excessive Wnt/β-catenin signaling contributes to the aging of mesenchymal stem cells though the DNA damage response and the p53/p21 pathways [27]. Furthermore, chronic activation of Wnt signaling induces fibrosis, hypertrophy, and stem cell senescence in aged heart [28]. Cytokines and chemokines also serve as contributing factors to the development and progression of aging and age-related inflammatory diseases. Higher blood levels of C-reactive protein (CRP), IL-6, and TNF-α have been associated with an increased risk of osteoarthritis, dementia, sarcopenia, and aging [29]. Interestingly, miR-34a induces vascular smooth muscle cell senescence via sirtuin 1 downregulation and promotes the expression of pro-inflammatory secretory factors such as IL-1β, monocyte chemoattractant protein 1 (MCP1), and IL-6 [30]. In contrast, CR and its mimetics were shown to reduce cancer as well as the progression of inflammatory diseases such as diabetes, and neurodegeneration through the regulation of miRNA expression [31]. Therefore, miR-500-3p and miR-770-3p may similarly contribute to accelerate the age-related senescence of stem cells and proliferating cells as well as age-associated inflammatory diseases. Many studies have revealed that miRNAs-containing microvesicles can transfer part of their genetic information to selected target cells via paracrine/endocrine signals, thus influencing physiological and pathological processes [3]. Therefore, further studies are required to clarify the cells or tissues of origin for miR-500-3p and miR-770-3p delivering exosomes and the detailed role of these two miRNAs in the aging process.

CR is the most useful dietary intervention known to delay aging and modulates the gene expression patterns which are altered with aging [10]. Dhahbi et al., (2013) showed that 18 circulating miRNAs that increased with aging are decreased by CR, and that these indicated miRNAs are involved in metabolic changes that occur with aging [32]. According to our recent result, short-term CR (4 weeks) induced epigenetic changes through amelioration of age-related methylation changes in promoters region in old rats [12]. Therefore, modulation of exosomal miR-500-3p and miR-770-3p by CR may underlie the anti-aging effects.

In summary, we have identified exosomal miRNAs are modulated by aging and CR, and examined their role during aging. In particular, we found that the expression of miR-500-3p and miR-770-3p was significantly increased with aging, whereas these were decreased by CR. Furthermore, predicted biological function of both miRNAs was associated with Wnt/chemokines and cytokines-related inflammatory signaling pathways and transcriptional regulation. Therefore, exosomal miR-500-3p and miR-770-3p could be potential biomarkers candidates to study aging.

## MATERIALS AND METHODS

### Animals

Male Sprague-Dawley rats, 7 (young) and 22 (old) months old, were obtained from Samtako (Osan, Korea). As reported previously [33], rats were housed individually in polycarbonate cages with wood chip bedding, maintained in an air-conditioned animal room (temperature: 24°C, relative humidity: 55 ± 5%) with 12 h light/dark cycle, and ad libitum (AL) access to feed and tap water. Each group included 6 rats. The old group was divided into two groups; control old rats were fed ad libitum, and the other experimental group was fed a CR diet for 4 weeks (old-CR) with 40% reduction of AL based on food consumption data collected from a preliminary study. After 4 weeks, 7 and 22 month-old rats were anesthetized. To obtain serum samples, blood was drawn and allowed to clot at room temperature for 30 min, and centrifuged at 2,500 × *g* at 4°C for 15 min. The serum was collected, and stored at −80°C until further analyses. The animal study was designed by the Aging Tissue Bank and approved by the Pusan National University-Institutional Animal Care and Use Committee (Approval Number PNU-2014-0601).

### Isolation of exosomes from rat serum

Serum exosomes were isolated using the Exiqon reagent (Takara Bio Inc., Shiga, Japan) following the manufacturer’s protocol. Briefly, serum was centrifuged at 10,000 × *g* for 5 min at 4°C to remove cell debris and then was filtered using 0.22 µm pore size syringe filter. The filtered serum (500 µl) was incubated overnight at 4°C with precipitation buffer. The mixture was centrifuged at 1,500 × *g* for 30 min and the supernatant was removed completely. The pelleted fraction was re-suspended by vortexing in 300 µl of resuspension buffer.

### Morphology analysis of exosomes using TEM

Exosomes suspended in PBS were loaded onto glow-discharged carbon-coated copper grids. After protein adsorption for 1 min, grids were stained with 1% (w/v) uranyl acetate for 1 min. The results were recorded using H-7600 TEM (Hitachi, Japan) at an acceleration voltage of 80 kV.

### DLS measurement of isolated exosomes

Size distribution of isolated exosomes was analyzed through DLS using Zetasizer Nano ZS (Malvern Instruments, Ltd., UK). The exosome size was analyzed through number percent (z-average) at a fixed angle of 173 at 25°C using software of the instrument.

### Western blot analysis of exosomes

Purified exosomes were treated with RIPA Buffer (50 mM Tris-HCl pH 7.4, 150 mM NaCl, 0.5 % sodium deoxycholate, 1 % NP-40, and 0.1 % sodium dodecyl sulfate) containing 1 mM phenlymethylsulfonyl fluoride, and protease inhibitor cocktail (Sigma-Aldrich, Saint Louis, MO, USA). Same volume from each sample was separated on 10 % SDS-PAGE gel, transferred to polyvinylidene fluoride membrane, and blotted with an antibody against CD63 and Alix (Santa Cruz Biotechnology, Santa Cruz, CA).

### Exosomal small RNA extraction

Exosomal small RNA was extracted using the Hybrid-R™ miRNA purification kit (GeneAll, Seoul, Korea) according to manufacturer’s instructions. Briefly, the isolated exosome was mixed with RiboEx solution, vortexed for 15 sec, and incubated at room temperature for 5 min. Then, 10 fmol of synthetic *C. elegans* miR-54 (Bioneer Inc., Daejeon, Korea) was spiked into the mixture as a normalization control. From this step, the manufacturers’ protocols were followed for RNA extraction. RNA concentration was determined using a NanoDrop spectrophotometer (Thermo Fisher Scientific Inc., Waltham, MA, USA) and Agilent 2100 Bioanalyzer (Agilent Technologies, Santa Clara, CA).

### Small RNA next-generation sequencing and bioinformatics analysis

We performed next generation sequencing of small RNA derived from young and old rats. Of 6 samples in each group, 2 randomly selected samples were pooled so that the total sample number (n) per group was three. That is, 3 samples each from young and old rats were used for small RNA sequencing. An RNA sequencing library was generated using the Nextflex Small RNA Seq Kit V3 (Bioscientific, TX, USA) according to the user manual. Briefly, small RNA library preparation involves ligating randomized adapters directly onto the 3′ and 5′ ends of RNA in two separate steps, followed by reverse transcription. After gel-free size selection and adapt-dimer reduction of cDNA, PCR amplification was performed, followed by clean-up and quality control. The HiSeq2500 platform was utilized to generate 50 bp single-end sequencing reads (Illumina, CA, USA). Cutadapter 1.11 was applied for data filtering to remove adapters and low quality sequences. For miRNA analysis, the miARma-Seq pipeline with the miRNA analysis workflow was utilized according to the user manual [34]. The workflow of miRNA analysis with miARma-Seq consisted with alignment with Bowtie2 [35] and entity quantification with FeatureCounts [36] of reads. The cutoff criteria was 3-fold increase or decrease in expression with significance (*P* value < 0.05) in the differential expression analysis of miRNA. Targeted genes for each age-altered miRNA were predicted using the web-based software, TargetScan. We obtained gene ontology from the PANTHER database.

### cDNA synthesis and qRT-PCR

To perform cDNA synthesis, the isolated miRNAs were subjected to poly (A) tailing, where 1 μg of RNA was polyadenylated using 5 U of poly (A) polymerase (Ambion Inc., Austin, TX). The reactions were performed at 37°C for 1 h. Reverse transcription of the poly (A) tailed miRNAs was performed using M-MLV reverse transcriptase (Invitrogen, Carlsbad, CA) following the manufacturer’s instructions. A solution (9.5 μl) including 1 μg of the poly (A) tailed miRNAs and 1 μl of 10 mM RT linker sequence (CTGTGAATGCTGCGACTACGA-18 dTs) was incubated at 65°C for 5 min to remove any secondary structures. Next, 5 μl of 10 mM dNTP mix, 4 μl of 5 × RT buffer (including 250 mM Tris–HCl, 375 mM KCl, 15 mM MgCl_2_, and 50 mM DTT), 0.5 μl of RNase inhibitor, and 1 μl of M-MLV reverse transcriptase were added. The reaction was then incubated at 42°C for 90 min. After incubation, the reaction was allowed to proceed at 94°C for 2 min. The synthesized cDNA was used as a template for qRT-PCR, which was performed using SYBR green real-time master mix (Bioline, London, UK). qRT-PCR and data analyses were performed using the CFX Connect System (Bio-Rad Laboratories Inc., Hercules, CA). The following sense primers were used in this study: rno-miR-500-3p, 5′-AATGCACCTGGGCAAGGGTTCA-3′; rno-miR-770-3p, 5′- GTGGGCCTGACGTGGAG-3′; rno-miR-450a-5p, 5′-TTTTGCGATGTGTTCCTAATGT-3′; rno-miR-196c-3p, 5′- ACAACAACACCAAACCACCTGA-3′; *Cel-*miR-54, 5′- UACCCGUAAUCUUCAUAA -UCCGAG -3′. All amplifications were carried out using the antisense primer (universal primer) 5′-CTGTGAATGCTGCGACTACGAT-3′.

### Statistical analysis

Values are shown as mean ± S.E. Analyses were performed using GraphPad Prism 5 (GraphPad software, La Jolla, CA, USA). Statistical significance of the differences between multiple tests was determined by a one-factor analysis of variance (ANOVA) followed by Bonferroni’s multiple comparison test. Values with *P* < 0.05 were considered statistically significant. The ROC curve and AUC were analyzed to assess the possibility as candidates for aging.

## SUPPLEMENTARY MATERIALS FIGURES


