# Supplementary material for: The involvement of serum exosomal miR-500-3p and miR-770-3p in aging: modulation by calorie restriction
Source: Oncotarget. 2017 Dec 24;9(5):5578–87. doi: 10.18632/oncotarget.23651 (PMC5814159; doi:10.18632/oncotarget.23651)
Supplement: Supplementary file 1 [file oncotarget-09-5578-s001.pdf]

# The involvement of serum exosomal miR-500-3p and miR-770-3p in aging: modulation by calorie restriction

## SUPPLEMENTARY MATERIALS

### Serum miRNA purification and cDNA synthesis

miRNA was isolated from 200 µl of serum from young (7-month), old (22-month), and old-CR rats using serum exosomal miRNA purification kit (Genolution, Inc., Seoul, Korea) according to manufacturer's instructions. Briefly, serum was mixed with miRNA extraction solution that was provided by Genolution, vortexed for 20 sec, and then 10 fmol of synthetic *C. elegans* miR-54 (Bionneer Inc) was spiked into the mixture as a normalized control. From this step, the manufacturers' protocols were followed for serum miRNA extraction. Isolated miRNA was polyadenylated and then reverse transcribed with primer with RT linker sequence.

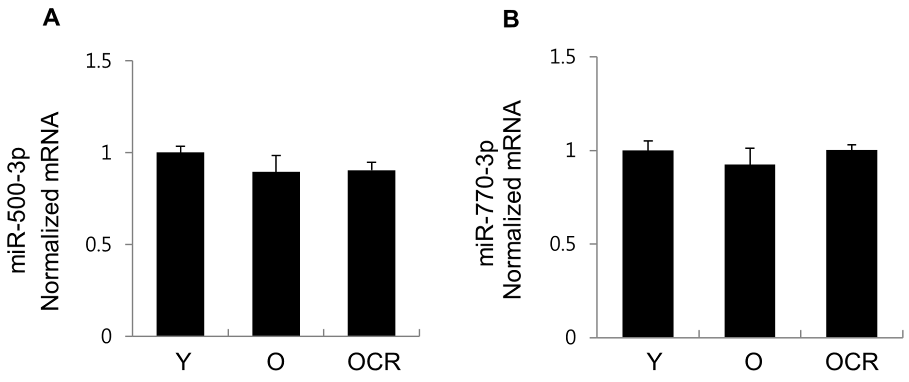


**Supplementary Figure 1: Expression of miR-500-3p and miR-770-3p in the serum of young, old and old-CR rats.** qRT-PCR was used to investigate the expression of miR-500-3p (**A**) and miR-770-3p (**B**). The obtained values were normalized to cel-miR-54 as an internal control. Each value is the mean ± S.E. (n = 6). Y, young; O, old; OCR, old-CR

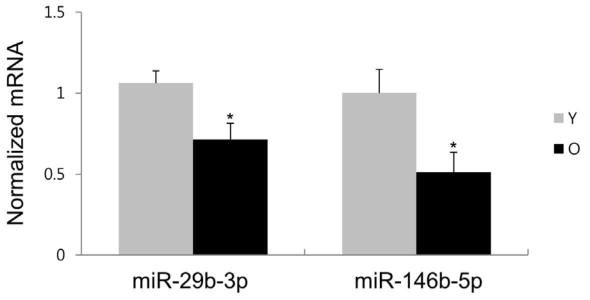


**Supplementary Figure 2: Expression of miR-29b-3p and miR-142-5p in the serum of young and old rats.** qRT-PCR was used to investigate the expression of miR-29b-3p and miR-142-5p. The obtained values were normalized to cel-miR-54 as an internal control. Each value is the mean ± S.E. *$P < 0.05$ vs. young rats group (n = 6). Y, young; O, old
